# An analysis of the relationship between dietary pattern changes and temporomandibular joint inflammation in diabetic rats

**DOI:** 10.34172/joddd.2023.40713

**Published:** 2023-12-30

**Authors:** Seyed Amir Abas Noorbakhsh, Mehrad Rafiei, Marzieh Hosseinabadi, Amin Amirkafi, Mostafa Sadeghi, Ali Peimani

**Affiliations:** ^1^Student Research Committee, Rafsanjan University of Medical Sciences, Rafsanjan, Iran; ^2^Dentist, Private Practice, Shiraz, Iran; ^3^Department of Operative Dentistry, School of Dentistry, Rafsanjan University of Medical Sciences, Rafsanjan, Iran; ^4^Department of Oral and Maxillofacial Surgery, School of Dentistry, Rafsanjan University of Medical Sciences, Rafsanjan, Iran

**Keywords:** Diabetes, Dietary pattern, Inflammation, TMJ

## Abstract

**Background.:**

The temporomandibular joint (TMJ) is the most commonly used joint in the human body. Recent studies have shown pathologic relationships between inflammation, diabetes, and musculoskeletal disorders (MSDs). Chewing disorder is a significant sign of dysfunction in the masticatory system. This study investigated dietary pattern changes in response to TMJ inflammation in diabetic rats.

**Methods.:**

This experimental study was carried out on 30 male rats. The rats were fed concentrated 20-mg dietary tablets. Complete Freund’s adjuvant (CFA) was used to induce TMJ inflammation and streptozotocin (STZ) was used to induce diabetes. The animals were randomly divided into three groups (n=10), including group I (CFA+STZ), group II (healthy rats+CFA), and group III (healthy rats, no injection). Parameters such as overall food intake, food intake duration, food intake frequency, and the interval between meals were recorded in a checklist and analyzed by Mann-Whitney and Kruskal-Wallis tests (*P*<0.05).

**Results.:**

The results showed no significant difference between groups in overall food intake and food intake frequency on days 0 and 1, but this difference was significant from day 2 to day 7. Regarding the time and end of food intake, there was a significant difference between the three groups from day 1 to day 7, but this difference was not significant on day zero.

**Conclusion.:**

Dietary pattern changes were similar in the diabetic TMJ inflammation and TMJ inflammation groups. These changes can be used as a behavioral marker for TMJ inflammation in rats.

## Introduction

 The temporomandibular joint (TMJ) is the most commonly used joint in the human body.^1^ TMJ may undergo inflammation, trauma, infection, and congenital and advanced illnesses like other joints. However, the most prevalent impairment of TMJ and the masticatory system is functional disorders associated with pain.^2^

 Approximately 5%‒6% of the world’s population suffers from painful experiences such as temporomandibular disorders (TMDs).^1^ TMD involves behavioral and biological changes, especially when followed by inflammation.^2-4^ TMJ inflammation can alter behavioral responses such as food intake and the release of stress hormones.^5^ It is conceivable that masticatory patterns will change during pain and joint inflammation.^2-5^ Altering the masticatory pattern to prevent pain limits the dietary choice.^3^ Chewing disorder is an objective sign of masticatory system dysfunction.^3,6^ Harper et al^6^ reported that dietary patterns could change following TMJ inflammation.

 Diabetes is followed by musculoskeletal disorders (MSDs), which can be caused by joint inflammation.^3,5^ Verhulst et al^7^ reported a relationship between diabetes and TMJ disorder and inflammation. Recent studies have shown pathologic associations between inflammation, diabetes, and atherosclerosis. The most important mediators and indicators of this mechanism include TNF-α, IL-6, calcitonin gene-related peptide (CGRP), and cytokines like TNF-α and IL-6 increase in patients with type II diabetes.^8-10^ Type II diabetes mellitus, one of the most common metabolic disorders, is caused by a combination of two primary factors: defective insulin secretion by pancreatic β-cells and the inability of insulin-sensitive tissues to respond appropriately to insulin. Insulin resistance, the common underlying abnormality, results from an imbalance between energy intake and expenditure, favoring nutrient-storage pathways, which evolved to maximize energy utilization and preserve adequate substrate supply to the brain. Initially, dysfunction of white adipose tissue and circulating metabolites modulate tissue communication and insulin signaling. However, when the energy imbalance is chronic, mechanisms such as inflammatory pathways accelerate these abnormalities.^11^

 Analysis of dietary patterns can be a non-destructive biological marker to study TMJ in an animal model.^12^ Changing the dietary pattern, such as changing the duration of food intake, is associated with inflammatory parameters such as external swelling, CGRP in the trigeminal ganglion, and level of substance P.^13,14^

 Complete Freund’s adjuvant (CFA) is a solution of antigens emulsified in mineral oil and consists of inactivated and dried mycobacteria. CFA injection is simple and reproducibly induces TMJ inflammation.^15^

 Despite the evidence of the association between diabetes and joint inflammation, including TMJ, and dietary pattern changes after TMJ inflammation in previous studies, no study has specifically investigated the changes in dietary patterns in diabetic mice with TMD. Therefore, no strong evidence can be provided on this subject. Additionally, this study compares the dietary pattern changes between two groups of diabetic and non-diabetic rats with TMJ inflammation and a control group. This comparison can examine the difference in the impact of diabetes on dietary pattern changes. Also, if this relationship exists, dietary pattern changes can be investigated as a possible parameter for TMJ disorders.

## Methods

**Figure 1:**
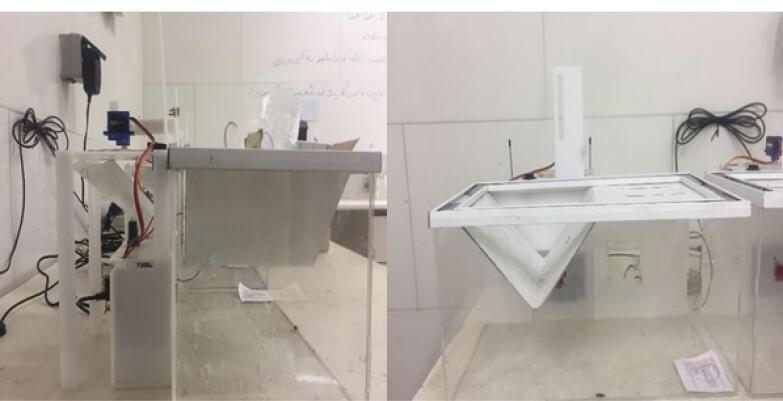
Smart feeding system

 This experimental study placed 30 Sprague-Dawley rats with a 220-250-g weight range and equal age in cages equipped with a smart feeding system (Figure 1). The animals were kept in standard conditions in a 12:12 light-dark (LD) cycle in a room with controlled temperature (23 °C) and sound insulation. They were fed concentrated 20-mg food tablets (Complete, Bruges, Belgium) with free access to water. The rats were placed in specially designed cages three days before the experiment to get used to the new environment and learn to take their food through the smart feeding system.

 The designed cages were made of 3-mm Plexiglas and chloroform solvent, with a lever installed. The rats pressed the lever to get their food through a window by a feeding system installed within the cage (Figure 1). The feeding system included a food source, a tube, and a touch-sensitive food cup. When the rats took their food through the window by touching the food cup, a message was sent to the operator by a reporting system, including the date, the accurate time, and the number of food tablets consumed.

 The recorded reports determined the time of food intake, overall food intake, frequency, and the interval between meals. The end of food intake was not completely defined in rats; therefore, three separate periods (5, 10, and 15 minutes) were considered for the end of food intake to measure the duration of food intake.^6^

 The reporting system consisted of a remote control unit (AM 643, Tak Electric, Tehran, Iran) with four outputs to report four cages. Each output was connected to a touch-sensitive sensor. One day before the experiment, the rats’ food intake level, water intake level, and weight were measured. CFA (Sigma, St. Louis, America) was used to induce TMJ inflammation, and streptozotocin (STZ) (Sigma) was used to induce diabetes.

 The samples were randomly divided into three groups, with 10 in each group. The first group received CFA plus STZ, the second group received CFA, and the third group received nothing (control group). One week before placing the rats in cages, the STZ dissolved in 0.1% citrate buffer (55 mg/kg) was administered intraperitoneally. One week after STZ administration, blood glucose was measured by a glucometer (Roche, Mannheim, Germany) to confirm that the rats were diabetic. Blood glucose above 250 mg/dL confirmed diabetes. Blood glucose was tested once during the experiment,^3^ and rats with lower blood glucose were excluded from the study.

 On the day of the experiment, the rats were anesthetized intraperitoneally after aspiration with ketamine (52 mg/kg) (Yasin Daru, Tehran, Iran) and xylazine (0.9 mg/kg) (Yasin Daru), which is 60% of normal dosage for anesthesia, using an insulin syringe (Ava Pezeshk, Tehran, Iran). A bilateral CFA injection into TMJ was performed. In groups I and II, 30 μg CFA in a 6-μL volume was injected. In the base group, no injection was performed. Dietary pattern changes were recorded one day before bilateral TMJ injection and seven days after injection.^6^

 The overall food intake, food intake duration, food intake frequency, and the interval between meals were recorded in a checklist.

 Data were statistically analyzed using SPSS 21 (SPSS Inc, Chicago, IL, USA). The normal distribution of the data was not confirmed using the Shapiro-Wilk test (*P* < 0.05). Therefore, Kruskal-Wallis and Mann-Whitney U tests were used. Quantitative data were reported as mean and standard deviation (mean ± SD), and qualitative data were reported as numbers (%). A *P* value of < 0.05 was considered statistically significant.

## Results

**Figure 2:**
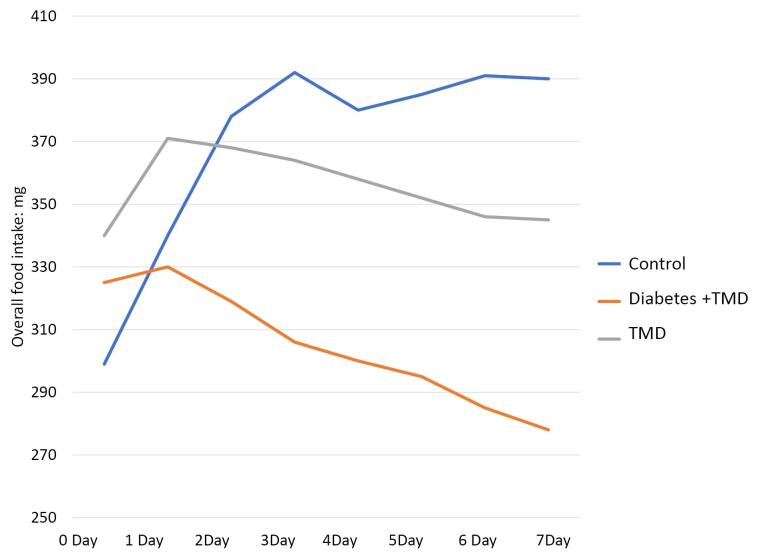
Comparison of overall daily food intake between different study groups

 Animals with equal weight and age were included in this study, so they were not significantly different. The overall food intake (mg/d) is shown in Figure 2. The comparison of the overall food intake between the three groups from day three to day seven after induction of inflammation showed a significant difference (*P* < 0.05). There was also a significant difference between the diabetic and TMJ inflammation groups from day 1 to day 7 after induction of inflammation (*P* < 0.05). Furthermore, there was a significant difference between the control and TMJ inflammation groups from day 2 to day 7 after the induction of inflammation (*P* < 0.05). Moreover, there was a significant difference between the diabetic and TMJ inflammation groups from day 2 to day 7 after induction of inflammation (*P* < 0.05).

**Figure 3:**
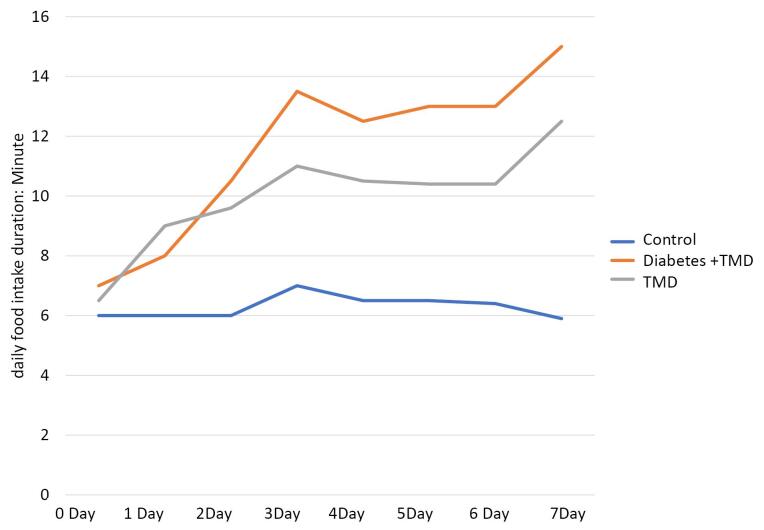
Comparison of daily food intake durations between different study groups

 The duration of food intake (day/min) is depicted in Figure 3. A comparison of food intake duration showed a significant difference between the three groups on day 1 (*P* < 0.05). After inducing inflammation, no significant difference was found on days 0 to 2. However, this difference was significant between the diabetic and TMJ inflammation groups from day 3 to day 7 after the induction of inflammation (*P* < 0.05).

 The results showed a significant difference between TMJ inflammation and control groups on days 1 to 7 after inducing inflammation (*P* < 0.05). Furthermore, a significant difference was found between the control and diabetic TMJ inflammation groups from day 1 after the induction of inflammation (*P* < 0.05).

**Figure 4:**
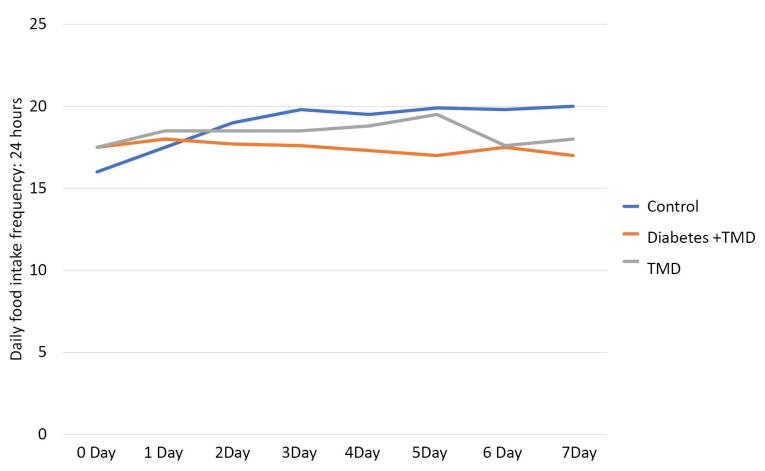
Comparison of daily food intake frequencies between different study groups

 The frequency of daily food intake is shown in Figure 4. The results of the Kruskal-Wallis test showed a significant difference between the three groups from day 2 (*P* < 0.05). This comparison also showed a significant difference between diabetic and TMJ inflammation groups from day 2 to day 5 and on day 7 after the induction of inflammation (*P* < 0.05). There was also a significant difference between the control and TMJ inflammation groups from day 3 to day 7 after the induction of inflammation (*P* < 0.05) and between the diabetic, TMJ inflammation, and control groups from day 2 to day 7 after the induction of inflammation (*P* < 0.05).

**Figure 5:**
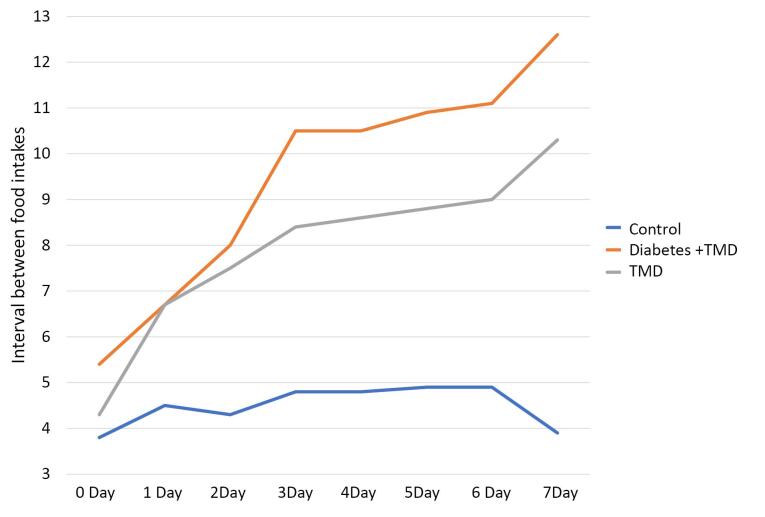
Comparison of the intervals between daily food intake between different study groups

 The interval between daily food intakes is indicated in Figure 5. The intervals between daily food intakes showed a significant difference between the three study groups (*P* < 0.05). This comparison also indicated a significant difference between the diabetic and TMJ inflammation groups from day 3 to day 7 after the induction of inflammation (*P* < 0.05). A significant difference was found between the control and TMJ inflammation groups 1‒7 days after the induction of inflammation (*P* < 0.05). Moreover, a significant difference was observed between the control and diabetic TMJ inflammation groups one day after the induction of inflammation (*P* < 0.05).

## Discussion

 Recently, pathologic relationships have been reported between inflammation, diabetes, and atherosclerosis.^16^ On the other hand, it has been demonstrated that diabetes could lead to MSDs.^16-18^ As a public health problem, MSDs contribute to pain and dysfunction, harming the healthcare system and disrupting the daily activities of individuals with diabetes through functional and activity limitations.^19^

 A common effect of diabetes is “stiffness,” a musculoskeletal problem caused by connective tissue damage that leads to limited joint movement.^20^

 The increased musculoskeletal problems in diabetics are related to neuropathy,^21^ vascular insufficiency,^22^ decreased insulin-like growth factor 1, obesity, accelerated osteoporosis, and a sedentary lifestyle.^23^

 The incidence of TMD among diabetic patients has been estimated to be 37%.^14^ TMJ disorders, especially when followed by inflammation, alter the chewing pattern^12,13^; changes in chewing behavior, frequency, and perception of mandibular limitation were observed in the presence of TMD.^24^ Hence, this study investigated the dietary pattern changes in response to TMJ inflammation in rats.

 This study showed that the presence of TMJ inflammation changed the chewing pattern. Furthermore, the induction of TMJ inflammation in diabetic rats increased the dietary pattern changes, i.e., food intake, food intake duration, and intervals between meals, which were significantly different between the control and the other experimental groups from the first day. The diabetic and TMJ inflammation groups showed significant differences from day three onward.

 Similar to the results of the present study, Li et al^25^ reported that the analysis of eating patterns has been suggested to be a noninvasive biological marker of TMJ inflammation and nociceptive behaviors. They used the automated behavioral classification system LABORAS to detect changes in eating patterns, including feeding duration and frequency, in mouse models of TMD. Their results indicated that TMD mouse models showed significantly increased eating duration and decreased eating frequency, suggesting that nociceptive behavior, likely caused by the movement of the TMJ during the chewing process, resulted in slowed and reduced eating.

 Harper et al^6^ reported a decrease in the overall food intake due to reduced food intake frequency.

 The present study showed similar results for the overall food intake in TMJ inflammation and diabetic TMJ inflammation groups. However, this reduction was not due to decreased food intake frequency. However, based on the reduced overall food intake, it can be concluded that the amount of half-eaten food by rats has been higher. Another study by Harper et al^13^ showed that the amount of food intake remained unchanged, but the length of food intake increased. The duration of food intake in the present study increased in both TMJ inflammation and diabetic TMJ inflammation groups.

 Kramer et al^14^ evaluated TMJ inflammation in rats and two mice. They indicated that CFA injection did not significantly change the frequency of food intake, which is consistent with the present study. According to the results of Kramer et al, 20 mg of food is considered a big size for a rat. If the size of food is an important factor in food intake duration, the length of food intake will increase, according to which the present study showed similar results. However, the duration of food intake was reduced in the study of Kramer et al, which can be due to another factor affecting the duration of food intake in terms of the exact injection location. The TMJ is much smaller in mice than in rats, so finding the exact location of injection is difficult, which can influence the response induced by CFA injection.

 Kerins et al^3^ investigated the dietary patterns in an animal model to determine TMJ pain and inflammation. They also evaluated the efficacy of dexamethasone use in treating TMJ inflammation and pain due to CFA injection. In this study, the overall food intake decreased in the group with CFA injection. However, the duration of food intake in the study of Kerins et al was normal and in contrast with the results of the present study, which can be due to the smaller size of food intake than that of the present study and higher frequency of food intake. In another study, Kerins et al^12^ investigated the analgesic effects of ibuprofen on dietary pattern changes and TMJ inflammation. They reported that food intake duration increased in the group receiving only CFA, but the results were normal in the group receiving ibuprofen. Similar to the present study, this study showed that dietary pattern changes could be used as a behavioral marker for TMJ pain and inflammation.

 According to this study and other studies,^3,6,18^ diabetes and TMJ inflammation can significantly affect the dietary pattern of rats. The present study indicated that dietary pattern changes in the diabetic TMJ inflammation group were the same as those of the TMJ inflammation group. These changes can be used as a behavioral marker for TMJ inflammation in rats. Moreover, further research on human samples is suggested in keeping with ethical principles. Physicians and dentists should also regard this issue as one of the main concerns of these patients to promote their living standards.

 A limitation of this study was the lack of accurate recording of the weight of rats before and after the study. Another limitation was the absence of pathologic analyses to study the amount of inflammation. Hence, future studies are suggested to record the weight of rats before and after the study to be taken as a variable. Further studies are also recommended to perform pathologic tests to evaluate the inflammatory cytokines to confirm inflammation. It is also suggested that another group be added to the samples and that the effect of diabetes on dietary pattern changes be evaluated separately because the interval between food intakes increased in the present study. On the other hand, the interval between meals was expected to reduce in diabetic rats. However, this study did not determine whether this increase was due to diabetes or inflammation.

## Conclusion

 The present study showed that dietary pattern changes were similar in the diabetic TMJ inflammation and TMJ inflammation groups and were different from TMJ inflammation-free rats. These changes can be used as a behavioral marker for TMJ inflammation in rats.

## Acknowledgments

 The authors wish to thank Sina Baghaeifar for his valuable contribution to designing and constructing the Smart feeding system.

## Competing Interests

 No author has any possible conflict of interests.

## Ethical Approval

 The present study was carried out according to the criteria of the Helsinki Declaration for medical research protocols and was approved by the Ethics Committee of the School of Dentistry, Rafsanjan University of Medical Sciences (Ethical code: IR.RUMS.REC.1401.098).

## Funding

 The research was funded by the Rafsanjan University of Medical Sciences.

## References

[1] Yadav S, Yang Y, Dutra EH, Robinson JL, Wadhwa S (2018). Temporomandibular joint disorders in older adults. J Am Geriatr Soc.

[2] Salamon NM, Casselman JW (2020). Temporomandibular joint disorders: a pictorial review. Semin Musculoskelet Radiol.

[3] Kerins CA, Carlson DS, Hinton RJ, Hutchins B, Grogan DM, Marr K (2005). Specificity of meal pattern analysis as an animal model of determining temporomandibular joint inflammation/pain. Int J Oral Maxillofac Surg.

[4] Murphy MK, MacBarb RF, Wong ME, Athanasiou KA (2013). Temporomandibular disorders: a review of etiology, clinical management, and tissue engineering strategies. Int J Oral Maxillofac Implants.

[5] Morel M, Ruscitto A, Pylawka S, Reeve G, Embree MC (2019). Extracellular matrix turnover and inflammation in chemically-induced TMJ arthritis mouse models. PLoS One.

[6] Harper RP, Brown CM, Triplett MM, Villasenor A, Gatchel RJ (2000). Masticatory function in patients with juvenile rheumatoid arthritis. Pediatr Dent.

[7] Verhulst MJL, Loos BG, Gerdes VE, Teeuw WJ (2019). Evaluating all potential oral complications of diabetes mellitus. Front Endocrinol (Lausanne).

[8] Spranger J, Kroke A, Möhlig M, Hoffmann K, Bergmann MM, Ristow M (2003). Inflammatory cytokines and the risk to develop type 2 diabetes: results of the prospective population-based European Prospective Investigation into Cancer and Nutrition (EPIC)-Potsdam Study. Diabetes.

[9] Rodrigues KF, Pietrani NT, Bosco AA, Campos FM, Sandrim VC, Gomes KB (2017). IL-6, TNF-α, and IL-10 levels/polymorphisms and their association with type 2 diabetes mellitus and obesity in Brazilian individuals. Arch Endocrinol Metab.

[10] Naujokat H, Sengebusch A, Möller B, Wieker H, Açil Y, Wiltfang J (2019). Antigen-induced arthritis of the temporomandibular joint via repeated injections of bovine serum albumin in domestic pigs. J Craniomaxillofac Surg.

[11] Roden M, Shulman GI (2019). The integrative biology of type 2 diabetes. Nature.

[12] Kerins C, Carlson D, McIntosh J, Bellinger L (2004). A role for cyclooxygenase II inhibitors in modulating temporomandibular joint inflammation from a meal pattern analysis perspective. J Oral Maxillofac Surg.

[13] Harper RP, Kerins CA, McIntosh JE, Spears R, Bellinger LL (2001). Modulation of the inflammatory response in the rat TMJ with increasing doses of complete Freund's adjuvant. Osteoarthritis Cartilage.

[14] Kramer PR, Kerins CA, Schneiderman E, Bellinger LL (2010). Measuring persistent temporomandibular joint nociception in rats and two mice strains. Physiol Behav.

[15] Wang XD, Kou XX, Mao JJ, Gan YH, Zhou YH (2012). Sustained inflammation induces degeneration of the temporomandibular joint. J Dent Res.

[16] Rehling T, Bjørkman AD, Andersen MB, Ekholm O, Molsted S (2019). Diabetes is associated with musculoskeletal pain, osteoarthritis, osteoporosis, and rheumatoid arthritis. J Diabetes Res.

[17] Sözen T, Başaran N, Tınazlı M, Özışık L (2018). Musculoskeletal problems in diabetes mellitus. Eur J Rheumatol.

[18] Alabdali LA, Jaeken J, Dinant GJ, van den Akker M, Winkens B, Ottenheijm RP (2021). Prevalence of upper extremity musculoskeletal disorders in patients with type 2 diabetes in general practice. Medicines.

[19] Sultana SR, Das M, Faruque M, Mondal R, Nurani RN, Hossain M (2015). Musculoskeletal disorders among Bangladeshi type 2 diabetic subjects. SMU Med J.

[20] Ravindran Rajendran S, Bhansali A, Walia R, Dutta P, Bansal V, Shanmugasundar G (2011). Prevalence and pattern of hand soft-tissue changes in type 2 diabetes mellitus. Diabetes Metab.

[21] Ramchurn N, Mashamba C, Leitch E, Arutchelvam V, Narayanan K, Weaver J (2009). Upper limb musculoskeletal abnormalities and poor metabolic control in diabetes. Eur J Intern Med.

[22] Shah KM, Clark BR, McGill JB, Mueller MJ (2015). Upper extremity impairments, pain and disability in patients with diabetes mellitus. Physiotherapy.

[23] Vancea DM, Vancea JN, Pires MI, Reis MA, Moura RB, Dib SA (2009). Effect of frequency of physical exercise on glycemic control and body composition in type 2 diabetic patients. Arq Bras Cardiol.

[24] Ferreira CLP, Sforza C, Rusconi FM, Castelo PM, Bommarito S (2019). Masticatory behaviour and chewing difficulties in young adults with temporomandibular disorders. J Oral Rehabil.

[25] Li J, Ma K, Yi D, Oh CD, Chen D (2020). Nociceptive behavioural assessments in mouse models of temporomandibular joint disorders. Int J Oral Sci.

